# Exploring the therapeutic applications of metformin in articular cartilage repair: mechanistic insights and translational perspectives

**DOI:** 10.3389/fimmu.2026.1916957

**Published:** 2026-08-28

**Authors:** Pan Jin, Xiaoxue Xin, Wei Liu, Xiaochi Zhu, Zengkun Li, Xiuhong Cao, Ziying Meng, Xiao Tian, Jiaxin Chen, Lixue Zou, Fabiao Yu, Tongmeng Jiang

**Affiliations:** 1 The Joint Department of Orthopedics, The First Affiliated Hospital of Yangtze University, Jingzhou, Hubei, China; 2 Health Science Center, Yangtze University, Jingzhou, Hubei, China; 3 NHC Key Laboratory of Tropical Disease Control, Engineering Research Center for Hainan Bio-Smart Materials and Bio-Medical Devices, Key Laboratory of Hainan Functional Materials and Molecular Imaging, School of Life Sciences and Medical Technology, Hainan Medical University, Haikou, Hainan, China; 4 School of Intelligent Medicine and Technology (Big Data Research Center), UWE College, Hainan Medical University, Haikou, China

**Keywords:** cartilage, chondrocytes, metformin, osteoarthritis, rheumatoid arthritis

## Abstract

Articular cartilage is characterized by an avascular structure and limited intrinsic regenerative capacity. The most prevalent pathological conditions that lead to cartilage damage are osteoarthritis (OA) and rheumatoid arthritis (RA). In recent decades, cartilage homeostasis has been shown to be particularly vulnerable to metabolic disturbances, especially those induced by obesity and diabetes mellitus. Metformin, a first-line antidiabetic agent and emerging antiaging therapeutic, has garnered significant attention as a promising candidate for cartilage repair that can address the unmet clinical need for treating currently irreversible cartilage damage and associated pathologies such as OA and RA. This review synthesizes preclinical and clinical evidence to elucidate the multifaceted effects of metformin on cartilage homeostasis. Cellular and animal studies have demonstrated that metformin exerts chondroprotective effects through multiple complementary mechanisms, including the activation of AMPK/SIRT1-mediated autophagy, the suppression of NF-κB signaling, the inhibition of matrix-degrading enzymes, the reduction of oxidative stress and inflammatory mediators, the modulation of macrophage polarization toward an anti-inflammatory phenotype, and the attenuation of aberrant subchondral bone remodeling. Emerging clinical evidence suggests that metformin may attenuate OA progression and ameliorate clinical symptoms. However, further large-scale, prospective trials are warranted to confirm its translation potential. By integrating findings across experimental models and clinical observations, this review highlights the role of metformin in modulating chondrocyte metabolism, mitigating inflammatory cascades, and promoting cartilage repair, thereby offering novel therapeutic strategies for cartilage-related disorders.

## 1 Introduction

Metformin, derived from the natural plant *Galega officinalis*, has been extensively employed in clinical practice because of its superior antihyperglycemic efficacy and reduced adverse effects compared with those of insulin (1). Although metformin is widely utilized for treating type 2 diabetes mellitus (T2DM), its effects on other diseases and related mechanisms remain incompletely understood. With the expanded clinical application of metformin, researchers have identified beneficial therapeutic effects beyond diabetes management (2), such as those associated with polycystic ovary syndrome (3), gut microbiota modulation (4), neurological disorders (5), cardiovascular disorders (6), oncological conditions (7), ankylosing spondylitis (8), mitochondrial dysfunction (9), aging (10), and obesity (11). Notably, metabolic pathologies, including diabetes and obesity, demonstrate epidemiological associations with musculoskeletal disorders, contributing significantly to physical disability and substantial socioeconomic burden (12, 13). Convincing evidence indicates that the glucose-lowering effects of metformin result from synergistic actions of multiple pathways, including but not limited to the inhibition of hepatic gluconeogenesis and glycogenolysis, in addition to increased insulin sensitivity in the skeletal muscle and peripheral tissues, collectively promoting glucose uptake and utilization (12).

Articular cartilage, situated at the osseous interface within musculoskeletal tissues, undergoes circadian rhythm regulation by mechanical forces (14). The mechanical alterations induced by obesity and the diabetic joint microenvironment represent significant pathogenic factors that disrupt articular cartilage homeostasis (14, 15). Given its avascular and aneural nature, cartilage degradation constitutes an irreversible pathological process for which effective pharmacological interventions remain limited (16). Osteoarthritis (OA), a prototypical manifestation of cartilage injury, has garnered substantial global attention from researchers and orthopedic clinicians (16). Epidemiological analyses have demonstrated its considerable burden: a meta-analysis reported a symptomatic knee OA prevalence of 14.6% in China; the Korean National Health and Nutrition Examination Survey (NHANES) reported a weighted radiographic knee OA prevalence of 35.1% among adults aged ≥50 years, and The Chingford Study reported a 17.6% cumulative 5-year incidence of radiographic knee OA meeting the “typical” criteria in women aged 45–64 years (17). Statistics concerning the incidence of OA exhibit considerable international variation. However, it is widely established that OA is characterized by high prevalence and disability rates. Given that joint replacement is the primary therapeutic option only in advanced stages, early intervention for OA remains a principal research focus. Moreover, the contemporary understanding of OA pathogenesis increasingly recognizes aging as a central underlying etiological factor (16). Consequently, once damaged, cartilage has a limited capacity for self-repair, making pharmacological intervention a critical area of research, particularly for prevalent conditions such as OA.

Given the background of the application of metformin in the treatment of aging-related diseases and OA pathogenesis due to aging, the possibility of metformin for OA therapy deserves investigation. Neumann B et al. reported that the differentiation potential of oligodendrocyte progenitor cells (OPCs) decreases with advancing age in adult rodents. Pharmacological treatment with metformin can reverse this age-associated decline, restore the regenerative capacity of aged OPCs, and increase remyelination efficiency in aged animals following focal demyelination (5). A study by Yang Y et al. demonstrated that metformin elicits potent antiaging effects via activation of the antioxidant transcription factor Nrf2, thereby conferring neuroprotection in primates. This intervention enhances cognitive function, preserves structural integrity, and reverses age-related brain degeneration by approximately 6 years (10). Furthermore, extensive research has revealed that metformin delays aging through targeting key hallmarks of senescence, including the dysregulation of nutrient sensing, loss of proteostasis, mitochondrial dysfunction, altered intercellular communication, telomere attrition, genomic instability, epigenetic alterations, stem cell exhaustion, and cellular senescence (18–20). Therefore, targeting the fundamental processes of aging constitutes a promising strategic approach for future OA therapies.

Rheumatoid arthritis (RA) is a chronic systemic autoimmune disease characterized by erosive, symmetrical polyarthritis. Its core pathological features include synovitis and pannus formation, ultimately resulting in articular cartilage and bone destruction, joint deformity, and loss of joint function (21). Metformin has shown potential for treating RA mainly through its ability to modulate immune responses and reduce inflammation. Its mechanisms may include the suppression of proinflammatory cytokines, the inhibition of nuclear factor κB (NF-κB) signaling, and the promotion of autophagy, all of which help decrease joint damage and autoimmune activity (22). For example, Kim et al. designed a drug repositioning strategy utilizing metformin encapsulated in poly(lactic-co-glycolic acid) (PLGA) and evaluated its effects on RA models. Their results demonstrated that the nanodrug can enter target cells via macropinocytosis and clathrin-mediated endocytosis and has more potent anti-inflammatory properties than free metformin does (23).

Obesity is also a significant risk factor for cartilage degradation. Li D et al. investigated the interrelationship among metformin administration, obesity, and cartilage damage. These findings demonstrated that metformin protected against OA in high-fat diet (HFD)-fed mice. This protective mechanism is mediated by a decrease in leptin secretion from adipose tissue, concurrently suppressing the infiltration and proinflammatory polarization of synovial macrophages (24).

Articular cartilage injury, subchondral bone hyperplasia, and synovitis are frequently observed in OA, RA and other conditions characterized by cartilage defects or injuries. Consequently, addressing these pathologies remains a significant challenge in the pursuit of cartilage repair. Chondrocytes, osteoblasts, macrophages, and other key cellular components, including synovial fibroblasts, play pivotal roles in the pathogenesis of these diseases. The initiation, progression, and outcomes of cartilage repair are intimately linked to the activities of these cells. As a conventional therapeutic modality, pharmacological intervention is more widely accepted than surgical approaches are. Drug repurposing is a promising strategy that may offer a favorable benefit-risk profile for cartilage repair (25). Metformin, an established antihyperglycemic and antiobesity agent, has demonstrated efficacy in this therapeutic context. Literature retrieval was conducted in three electronic databases, namely PubMed, Web of Science and Embase, spanning the period from database establishment to December 2025. The core search terms applied in this work included “metformin”, “cartilage”, “osteoarthritis”, “chondrocyte” and “rheumatoid arthritis”. Eligible publications were defined as English-language peer-reviewed original research articles and review articles covering *in vitro* experiments, animal studies and human clinical studies, while conference abstracts, case reports, duplicate publications and studies without accessible full text were excluded. Two authors independently screened titles, abstracts and full texts sequentially to identify final eligible literature. This review systematically examines the effects of metformin on different types of articular cartilage damage across four domains, including *in vitro* cellular experiments, preclinical animal studies, clinical investigations, and bioinformatics analyses, with the aim of identifying novel directions for future research.

## 2 *In vitro* cellular experiments

*In vitro* culture systems, including monolayer cell assays and isolated cartilage explants, can exclude systemic confounding signals from neuroendocrine and immune networks, thereby enabling direct and accurate quantification of drug-specific cellular and molecular responses. For preliminary pharmacological screening and mechanistic dissection of candidate anti-OA agents represented by metformin, *in vitro* experimental platforms function as indispensable preclinical tools prior to *in vivo* animal validation. To systematically elucidate the multi-layered chondroprotective effects of metformin and reconcile heterogeneous observations across independent laboratories, the following section synthesizes existing *in vitro* evidence grouped by core functional pathways, conducts stratified discussion of mouse, rat and human experimental models, performs comparative analysis of conflicting results, and finally summarizes the established research consensus and outstanding research limitations.

### 2.1 *In vitro* evidence from mouse models

Primary mouse chondrocytes and the ATDC5 chondrogenic cell line are the most widely adopted low-cost screening models for deciphering the intracellular signaling cascades of metformin triggered by interleukin-1 beta (IL-1β)-induced inflammatory injury. Cumulative studies conducted in mouse models converge on adenosine monophosphate-activated protein kinase (AMPK) as the master upstream effector that coordinates mitochondrial homeostasis, autophagy, extracellular matrix (ECM) turnover, inflammatory suppression and lipid metabolism.

#### 2.1.1 AMPK-SIRT3-mediated mitophagy and mitochondrial protection

Li H et al. utilized real-time quantitative polymerase chain reaction (RT–qPCR) and western blot analysis to investigate the anabolic and anticatabolic effects of metformin on IL-1β-stimulated primary mouse chondrocytes and human cartilage explants. Findings from this study demonstrated that metformin protects against IL-1β-induced catabolism in both chondrocytes and cartilage explants via the activation of AMPK signaling (26). Wang C et al. demonstrated that IL-1β stimulation downregulates the expression of mitochondria-protective Sirtuin 3 (SIRT3) in mouse primary chondrocytes (27). Pharmacological blockade of SIRT3 via 3-TYP further impaired PTEN-induced putative kinase protein 1 (PINK1)/Parkin-dependent mitophagy, reduced the LC3II/LC3I ratio and the abundance of anabolic collagen type II (Col II), while upregulating the expression of catabolic matrix metalloproteinase-3 (MMP3) and MMP13. Metformin reverses this damaging phenotype by upregulating SIRT3 to restore mitophagic flux, which constitutes a critical mitochondrial rescue axis specific to mouse chondrocyte injury models (27).

#### 2.1.2 AMPK-SIRT1-autophagy regulatory axis

Parallel siRNA loss-of-function assays have confirmed that metformin-induced autophagy is entirely dependent on the AMPKα2-SIRT1 signaling pathway, and deletion of the AMPKα1 isoform exerts no regulatory effect on chondrocyte autophagy (28). Feng X et al. further demonstrated that in IL-1β-treated mouse chondrocytes and cartilage explants, metformin suppresses the senescence marker p16^INK4a^ and cartilage degenerative collagen type X (Col X), while upregulating anabolic collagen type II alpha 1 (Col2a1) and SRY-box transcription factor 9 (SOX9) (29).

#### 2.1.3 Downstream anti-catabolic, anti-inflammatory signaling branches

Through AMPK activation, metformin concurrently blocks two pivotal OA-driving pathways: the proinflammatory NF-κB cascade and β-catenin-mediated osteogenic transdifferentiation signaling. In ATDC5 cells, metformin attenuates IL-1β-triggered extracellular matrix (ECM) degradation, proinflammatory cytokine release and chondrocyte apoptosis through AMPK-dependent inhibition of NF-κB (30). In both ATDC5 and primary mouse chondrocytes, metformin suppresses Ser552 phosphorylation, nuclear translocation and the transcriptional activity of β-catenin, thereby preventing hypertrophic transformation of chondrocytes (31).

#### 2.1.4 Crosstalk with macrophages, lipid homeostasis and ferroptosis

Beyond the intrinsic chondroprotective effects of metformin, this compound exerts indirect anti-inflammatory activities by remodeling macrophage polarization in co-culture systems. *In vitro* conditioned medium assays have confirmed that metformin suppresses the secretion of proinflammatory mediators by M1 macrophages, attenuates the chemotaxis of M1 macrophages toward injured chondrocytes, and mitigates the secondary inflammatory activation of chondrocytes (32). Following bioinformatic prediction, validation conducted in mouse chondrocytes further identified a novel AMPK/ACC axis through which metformin rewires dysregulated lipid metabolism and attenuates chondrocyte ferroptosis, a newly characterized driver of cartilage degradation under inflammatory stimulation (33).

#### 2.1.5 Targeted nanocarrier delivery optimization in mouse chondrocyte platforms

The ferritin nanocage carrier CT-Fn/Met has been engineered to achieve specific binding to type II collagen to better target cartilage and deliver metformin. Compared with free metformin, this formulation exerts more potent suppression of OA catabolic markers and upregulates the expression of cartilage-specific matrix proteins in IL-1β-stimulated mouse chondrocytes, confirming the translational advantage of targeted local delivery (34).

### 2.2 *In vitro* assays using rat primary chondrocytes

Compared with mouse chondrocyte cultures, rat chondrocyte cultures feature superior stability for long-term investigations of pharmacotoxicology and age-related OA pathogenesis, rendering them an optimal research model for exploring chondrocyte senescence and lipid metabolic disorders. Two core lines of existing evidence center on biomaterial-based delivery systems and cholesterol metabolic regulation. First, the zwitterionic sulfobetaine-modified hyaluronic acid methacrylate hydrogel (Met@SBHA) synthesized via microfluidic technology enables electrostatic loading of metformin. In primary rat chondrocytes, Met@SBHA maintains cellular metabolic homeostasis and retards cellular senescence by inhibiting the inducible nitric oxide synthase (iNOS)/peroxynitrite (ONOO-)/P53 oxidative stress-senescence signaling cascade (35). Second, Xing H et al. have verified the dual regulatory role of the AMPK/SIRT1 pathway in cholesterol homeostasis: metformin activates this pathway to simultaneously inhibit *de novo* cholesterol synthesis and promote cholesterol efflux, thereby preventing chondrocyte damage mediated by lipid accumulation (36).

### 2.3 *In vitro* testing using human clinical cartilage specimens

Human primary chondrocytes isolated from end-stage OA patients can optimally recapitulate clinical pathological backgrounds of the disease. However, these models inevitably harbor heterogeneous patient-derived confounding variables, which result in divergent research outcomes relative to rodent-based experiments. Existing human chondrocyte studies are categorized into four core research domains including bulk chondrocyte response, senescent subpopulation-specific effects, biomaterial sustained delivery, and mesenchymal stem cell osteochondral differentiation research.

#### 2.3.1 Consistent protective phenotypes across most human chondrocyte studies

Numerous human chondrocyte-based experiments are consistent with conclusions derived from rodent studies regarding the anti-catabolic, anti-inflammatory and pro-autophagy effects of metformin. Na HS et al. reported that metformin suppresses MMP-1/3/13 and pyroptosis mediators phospho-mixed lineage kinase domain-like protein (p-MLKL) and caspase-1, while upregulating autophagy markers light chain 3IIB (LC3IIB), prostacyclin 62 (p62), lysosome-associated membrane protein 1 (LAMP1), and tissue inhibitors of metalloproteinases TIMP-1/3 to facilitate autophagosome-lysosome fusion (37). In human chondrocytes stimulated with IL-26, metformin blocks the toll-like receptor 4 (TLR4)-extracellular signal-regulated kinases 1/2 (ERK1/2)-c-Jun signaling pathway to attenuate the overexpression of cyclooxygenase-2 (COX-2), IL-6, and MMP-1 (38). Transfection assays have confirmed that miR-34a mediates metformin’s suppressive effect on senescence-associated P16, inflammatory IL-6 and catabolic MMP-13, accompanied by elevated expression of Col II and aggrecan (39). Sustained-release carriers, including the DNA supramolecular hydrogel MET@DSH and strontium alginate hydrogel loaded with metformin, potently inhibit oxidative stress, apoptosis, matrix degradation and inflammation in human chondrocytes, and drive macrophage polarization toward the anti-inflammatory M2 phenotype (40, 41). Biomimetic biphasic scaffolds co-loaded with metformin and kartogenin effectively support robust osteochondral differentiation of human mesenchymal stem cells *in vitro* (42).

#### 2.3.2 Contradictory experimental outcomes and systematic analysis of discrepancy sources

Two core inconsistent findings have been exclusively observed in human end-stage OA chondrocyte models, which contradict the results obtained from healthy rodent chondrocytes.

The first one is the divergent regulation of SOX9 transcription factor. Experiments on rodent chondrocytes consistently demonstrate that metformin upregulates SOX9 to promote chondrogenesis. However, Schadler P et al. observed the simultaneous downregulation of SOX9 and catabolic ADAM metallopeptidase with thrombospondin type 1 motif 5 (ADAMTS5) following metformin treatment in human chondrocytes harvested from knee arthroplasty patients (43). Human specimens harbor complex clinical covariates that are absent in standardized rodent models, including elevated BMI, limb malalignment, chronic proton pump inhibitor (PPI) administration and high systemic C-reactive protein (CRP)-mediated inflammation. These background pathological factors alter the basal transcriptional profile of chondrocytes and modify the downstream transcriptional output of metformin.

The second is the opposing effects on senescent chondrocyte viability. Yan S et al. and Xu L et al. independently confirmed that metformin alleviates senescence marker expression and cellular senescence in bulk human OA chondrocyte populations (39, 41). In contrast, Ro DH et al. specifically sorted DPP-4^+^ senescent chondrocyte subpopulations and found that metformin did not induce a significant viability difference between DPP-4^+^ senescent cells and DPP-4^-^ normal chondrocytes (44). The potential confounding factors maybe the differences in cellular subpopulation screening strategies. Most studies analyze mixed unsorted chondrocyte populations, while the work of Ro DH et al. isolated DPP-4-labeled senescent subclones with unique metabolic characteristics. Thus, the senolytic or senomorphic activity of metformin varies substantially across chondrocyte subpopulations. There are four generalized factors responsible for inter-study heterogeneity (1): Different experimental models such as healthy mouse/rat primary chondrocytes, ATDC5 cell lines, unsorted human end-stage OA chondrocytes, sorted DPP-4^+^ senescent human chondrocytes; (2) Different injury stimulations such as IL-1β, IL-26, lipid overload, oxidative senescence induction; (3) Different metformin administrations such as free drug versus hydrogel/nanocage carrier loading, differing drug concentrations and treatment durations; (4) Patient-specific confounders such as obesity, diabetes, chronic medication, joint deformity and systemic inflammatory status.

### 2.4 Synthesis of consensus and unresolved research gaps from *in vitro* metformin OA studies

#### 2.4.1 Consistent consensus across all preclinical *in vitro* models

Across mouse, rat, and human chondrocyte models, current evidence has converged on the unified core mechanisms underlying metformin’s action: (1) AMPK functions as the core upstream signaling hub that integrates multiple downstream pathways, including the SIRT1/SIRT3, NF-κB, Wnt/β-catenin, and AMPK/ACC lipid metabolism axes; (2) Consistent chondroprotective phenotypes have been reproducibly observed across studies: suppression of matrix-degrading enzymes of the MMP/ADAMTS families; upregulation of Col II, aggrecan, and chondrogenic SOX9 (in non-end-stage healthy chondrocytes); inhibition of M1 macrophage-mediated inflammation; attenuation of oxidative stress, ferroptosis and apoptosis; and restoration of autophagic/mitophagic flux; (3) Sustained-release biomaterial delivery systems enhance metformin’s chondroprotective efficacy compared with free drug administration.

#### 2.4.2 Unresolved critical research gaps

Also, there are some unsolved critical research gaps. First, compared with rodent studies, current human chondrocyte research remains insufficient, and contradictory observations derived from end-stage OA specimens lack mechanistic explanations. The response characteristics of specific senescent chondrocyte subpopulations require further clarification. Second, systematic comparisons of metformin’s dose-response relationships across different concentrations and treatment durations are rarely reported, especially regarding metformin’s regulatory effects on senescence and ferroptosis. Third, the interactive regulatory network connecting lipid homeostasis, ferroptosis, mitochondrial mitophagy and cellular senescence has not been fully delineated. Forth, complex multi-cell co-culture systems (chondrocyte-synoviocyte-adipocyte) that recapitulate the native joint microenvironment remain scarce in current research. Fifth, stratified mechanistic research on chondrocytes derived from obese and diabetic OA patients remains insufficient to explain the heterogeneity observed in clinical populations.

To facilitate intuitive comparison of metformin’s *in vitro* effects across different models and experimental readouts, all representative studies summarized above are compiled in **Table 1**. Despite remaining controversies, the preponderance of available *in vitro* data confirms that metformin counteracts cartilage damage induced by glucose, growth factors, insulin and amino acids. Via modulation of the AMPK/mTOR signaling pathway, metformin rewires lymphocyte immunometabolism and balances pro- and anti-inflammatory mediators within the joint tissue microenvironment (45).

**Table 1:** Effects of metformin on injured chondrocyte models: *In vitro* experimental findings.

Sources	*In vitro* models	Effects of metformin	Mechanisms of metformin	Refs
6~8-week-old C57BL/6 male mice	Articular chondrocytes stimulated with IL-1β	Suppressing IL-1β-induced oxidative and osteoarthritis-like inflammatory changes	Upregulating SIRT3-mediated PINK1/Parkin-dependent mitophagy	(27)
Newborn mice within 3 days; tibial plateau cartilages of 8-week-old mice	Articular chondrocytes and cartilage explants induced with IL-1β	Reducing matrix-degrading enzyme production and promoting chondrocyte synthesis	Activating AMPK and suppressing mTORC1	(29)
Femoral condyles and tibial plateaus of postnatal Day 3–4 C57BL/6 mice; Femoral condyles of total knee arthroplasty patients with a 2-mm biopsy punch.	Articular chondrocytes and cartilage explants treated with IL-1β	Protecting against IL-1β-driven catabolism in chondrocytes and cartilage explants	AMPK activation	(26)
7-day-old C57BL6/J mice	Articular chondrocytes induced with IL-1β	Decreasing MMP-1, MMP-13, IL6; upregulating COL2A1	Targeting to collagen type II produced by chondrocytes	(34)
6~8-week-old male C57BL/6 mice	Articular chondrocytes stimulated with IL-1β	Increasing autophagy and decreasing catabolism and apoptosis	Activating AMPK/SIRT 1-mediated autophagy in the primary cultured chondrocytes	(28)
Mouse ATDC5 chondrocyte cell line	Articular chondrocytes treated with IL-1β	Exerting anti-catabolic, anti-inflammatory, and anti-apoptotic effects in the IL-1β-induced ATDC5 chondrocytes	Regulation of the AMPK/NF-κB signaling pathway	(30)
ATDC5; primary human chondrocytes from the knee; mouse bone marrow cells (BMCs) from femurs and tibias of 12-week-old male C57BL/6 mice	ATDC5 and primary human chondrocytes were incubated with fat conditioned medium; BMCs were treated with M-CSF	Exerting a protective effect on chondrocytes, reducing the differentiation of mouse BMCs into bone marrow-derived macrophages (BMDMs), decreasing the M1/M2 macrophage ratio of BMDMs	Reducing leptin secretion; decreasing the M1/M2 ratio by activating AMPK signaling and inhibiting mTORC1 signaling	(24)
ATDC5; primary mouse knee chondrocytes from articular cartilage of 4-day-old neonatal mice	Transfected with AK mouse plus transforming or AKT2 or the TOP/FOP-flash reporter plasmid	Inhibiting β-catenin^S552^ phosphorylation and β-catenin signaling	AMPK activation	(31)
Primary articular chondrocytes were isolated from 5-day-old neonatal mice; BMCs were flushed out from the long bones of the mice; normal human synovium tissues were collected from injured in car accidents and underwent knee replacement surgery	Chondrocytes cultured with M1 macrophages conditional medium (CM) or IL-1β; BMCs were cultured in CM with M-CSF then stimulated with LPS and IFN-γ	Inhibiting macrophage-derived CM-induced chondrocyte degeneration; inhibiting inflammatory cytokine levels produced by M1 macrophages	Targeting PI3K/AKT and downstream mTOR, FoxO1, GSK3β and NF-κB pathways	(32)
Primary mouse chondrocytes were harvested from knee joint cartilage of 5-day-old C57BL/6J mice; human cartilage tissues were collected from OA patients who had undergone complete knee replacement surgery	Articular chondrocytes exposed to varying doses of IL-1β	Protecting chondrocytes affected by osteoarthritis, and alleviating the progression of osteoarthritis by regulating chondrocyte lipid metabolism and suppressing ferroptosis	Ameliorating chondrocyte ferroptosis sensitivity via the AMPK/acetyl-CoA carboxylase (ACC) signaling pathway	(33)
Mouse mononuclear macrophage line (J774); T cells were isolated from the spleens of 4-6-week-old male healthy DBA/1 mice and CIA models	J774 cells were stimulated by LPS or TNF-α to develop M1 phenotype	Decreasing the expression of pro-inflammatory cytokines (IL-1β, IL-6, and TNF-α), increasing the expression of anti-inflammatory cytokines (IL-10 and IL-4), and promoting the polarization of M1 to M2 macrophages	Unclear	(23)
1 month old Sprague–Dawley (SD) rats	Articular chondrocytes induced with insulin	Attenuating chondrocyte senescence, and orchestrating the metabolic equilibrium of chondrocytes	Alleviating chondrocyte senescence via iNOS/ONOO-/P53 axis	(35)
Newborn Wistar rats, patients undergoing knee or hip arthroplasty	Normal articular chondrocytes were treated with IL-1β; human OA chondrocytes were harvested from OA cartilage tissue	Inducing AMPK and SIRT1 activation, reversing cholesterol accumulation, decreasing cartilage matrix degradation	Reversing cholesterol accumulation via the AMPK/SIRT1 pathway	(36)
Femoral cartilage of female patients undergoing knee arthroplasty for end-stage knee OA	Primary human OA chondrocytes	Reducing ADAMTS5 and MMP1	May be through both AMPK dependent and AMPK-independent mechanisms	(43)
Cartilage of OA patients undergoing replacement arthroplasty or joint replacement surgery	Articular chondrocytes cultured with IL-1β	Decreasing the catabolic response of human OA chondrocytes, promoting autophagy in human chondrocytes	Regulating autophagolysosome via LAMP1 activation	(37)
Human cartilage of patients who underwent total knee replacement	Articular chondrocytes treated with IL-26	Attenuating the expression of COX-2, MMP-1, and IL-6	Via TLR4-extracellular signal-regulated protein ERK1/2-c-Jun signaling pathway	(38)
OA articular cartilage samples were aseptically harvested from femoral condyles and tibial plateaus of patients with OA at K/L grade of 3 or 4 who received total knee arthroplasty	Articular chondrocytes were transfected with miR-34a mimics, siRNA-SIRT1 or negative control	Attenuating chondrocytes senescence, promoting cell proliferation and viability of chondrocytes	Through microRNA-34a/SIRT1 pathway	(39)
All of the knee cartilage from knee joints was from patients with osteoarthritis who had undergone knee replacement; Monocyte macrophages (RAW264.7 cells)	Articular chondrocytes incubated with IL-1β, RAW 264.7 cells were stimulated with LPS for 24 h to induce the M1 subtype of macrophages.	Exerting antioxidant, anti-inflammatory, and pro-chondrocyte properties, inhibiting IL-1β-induced ECM degradation and apoptosis in chondrocytes	Polarizing the macrophages from the M1 type to the M2 type	(40)
hMSCs were collected from bone marrow aspirates, and mononuclear cells were isolated by Ficoll–Paque PLUS solution during total hip- and knee-joint arthroplasty surgery.	Not mentioned	Biphasic scaffold (BPS) loaded with two small-molecule drugs, KGN and metformin upregulated both osteogenic and chondrogenic gene expression, sulfated glycosaminoglycan (sGAG), osteo- and chondrogenic biomarker	The mechanism was not studied in this experiment.	(42)
Cartilage specimens were harvested from isolated knee joint bulks derived from one aged patient in late-stage OA who underwent total joint arthroplasty.	Not mentioned	Inhibiting senescence, inflammation, oxidative, and apoptosis in OA chondrocytes	May be related to autophagy and AMPK activation	(41)
Human OA cartilage from 10 patients undergoing total knee arthroplasty and non-OA cartilage from 10 patients who underwent tumor removal	Cultured in a chondrogenic medium	Does not affect the survival rate of DPP-4^-^ and DPP-4^+^ chondrocytes	The mechanism was not studied in this experiment.	(44)

## 3 Animal evidence for the effects of metformin in cartilage

*In vitro* cell culture systems enable precise control of individual experimental variables and direct mechanistic observation, yet they remain incapable of recapitulating the complex systemic crosstalk among circulation, immune cells, subchondral bone, adipose tissue and joint synovium under physiological or pathological conditions. Accordingly, *in vivo* whole-animal models constitute an indispensable translational bridge for validating the actual therapeutic efficacy, tissue distribution, systemic safety and multi-organ regulatory effects of metformin on osteoarthritic cartilage. This section systematically synthesizes all currently available *in vivo* evidence on metformin-mediated cartilage protection, categorizes relevant findings by animal species, and organizes them according to unified functional regulatory axes. Targeted comparative analyses of inconsistent experimental results from independent studies are performed, key confounding factors leading to heterogeneous outcomes are elucidated, and existing consensus and unresolved research gaps in the field are summarized and identified.

### 3.1 Mouse models of injured cartilage

Mouse models are the most widely adopted preclinical models for OA, owing to their low feeding cost, rapid reproduction, standardized surgical modeling procedures and well-established gene knockout technical systems. Cumulative *in vivo* studies on mouse models collectively confirm that metformin exerts multi-layered protective effects targeting chondrocytes, synovial macrophages, subchondral bone and systemic metabolic inflammation, with AMPK signaling serving as the core upstream regulatory hub. All relevant findings are integrated into six functional modules presented below.

#### 3.1.1 Core AMPK-dependent chondroprotective signaling cascades

Multiple mouse DMM/ACLT surgical OA models validated that metformin activates AMPK to orchestrate multiple downstream chondroprotective axes. Feng X et al. confirmed metformin alleviates cartilage degeneration and cellular senescence via AMPK/mTOR-mediated autophagy restoration (29). Wang C et al. further supplemented that AMPK/SIRT1 autophagy activation is required for metformin to attenuate cartilage matrix breakdown (28). Gene knockout experiments using AMPKα1-deficient mice verified that metformin’s inhibitory effects on synovial hyperplasia, osteophyte formation and OA pain perception are completely abrogated without functional AMPKα1. And AMPKα1 phosphorylation in cartilage and dorsal root ganglia is the necessary molecular mediator of metformin’s therapeutic activity (46). Additionally, metformin suppresses β-catenin Ser552 phosphorylation and nuclear translocation in OA cartilage to block chondrocyte hypertrophic transformation, acting as a downstream inhibitory branch of AMPK activation (31).

#### 3.1.2 Regulation of synovial macrophage polarization and intra-articular inflammatory microenvironment

Metformin alleviates joint inflammation primarily by reversing the M1 macrophage predominance in synovial tissue. In DMM-operated mice, synovial M1 macrophage infiltration is significantly elevated, while metformin suppresses M1 macrophage recruitment and pro-inflammatory cytokine secretion by inhibiting the PI3K/AKT signaling cascade, thereby mitigating secondary cartilage damage (32). Zou Z et al. further identified a chemotactic regulatory loop as ferroptotic chondrocytes secrete CCL2 to recruit synovial macrophages, while metformin blocks ferroptosis and interrupts this chemokine-mediated inflammatory cascade in ACLT mouse OA models (33). For cartilage injury concurrent with RA, PLGA-encapsulated metformin in combination with photothermal therapy effectively alleviates systemic and local joint inflammation in mouse RA models (23).

#### 3.1.3 Dual regulation of lipid metabolism and chondrocyte regulated cell death

In high-fat diet (HFD)-induced metabolic OA models, metformin corrects dysregulated lipid homeostasis, thereby suppressing two major chondrocyte death modalities including pyroptosis and ferroptosis. Yan J et al. reported that metformin inhibits NLRP3 inflammasome activation to reduce chondrocyte pyroptosis, reversing abnormal subchondral bone remodeling in DMM mice (47). In mouse OA models with erastin-induced ferroptosis, metformin restrains chondrocyte ferroptosis to block subchondral osteosclerosis and pathological angiogenesis, thereby preserving cartilage integrity (48). In HFD-driven metabolic OA, metformin reduces macrophage accumulation and prostaglandin synthesis in subchondral bone via inhibition of the COX-2-PGE2 signaling axis, thereby alleviating abnormal bone formation and joint pain (49). Li D et al. further confirmed that metformin reduces adipose leptin secretion, decreases chondrocyte apoptosis and matrix-degrading protease expression, and attenuates M1 macrophage infiltration in obese OA mice (24).

#### 3.1.4 Combined intervention with anti-osteoporotic drugs or physical exercise

Metformin exerts synergistic therapeutic effects when used in combination with alendronate or exercise. In collagenase-induced OA mice, co-administration of metformin and alendronate synergistically suppresses the RANK/RANKL bone resorption pathway, reduces serum levels of resistin and leptin, and attenuates cartilage degradation (50). Lambova SN et al. further verified that this combination regimen improves subchondral bone volume and sclerosis, downregulates the cartilage degradation markers CTX-II and COMP, and reduces pro-inflammatory visfatin levels (51). For OA induced by combined estrogen deficiency and obesity, co-intervention with metformin and exercise mitigates systemic inflammation and inhibits articular degeneration, with superior efficacy relative to single-agent monotherapy (52).

#### 3.1.5 Cartilage-targeted sustained delivery biomaterials for intra-articular administration

Oral metformin administration is characterized by low joint bioavailability, and direct intra-articular injection of the drug suffers from short intra-articular retention. A variety of engineered drug delivery carriers overcome this limitation via localized sustained drug release. The cartilage-targeting peptide-modified ferritin nanocage CT-Fn/Met achieves 3-week intra-articular retention, enhances chondrocyte uptake of the drug, and produces stronger therapeutic efficacy against OA compared with free metformin (34). Zwitterionic hydrogel microspheres Met@SBHA improve joint lubrication, delay chondrocyte senescence, and attenuate age-related OA in aged mice (35). The DNA supramolecular hydrogel MET@DSH enables stable local release of metformin to eliminate intra-articular inflammation and promote cartilage repair (40).

#### 3.1.6 Metabolic comorbidity-associated mouse OA models

Diabetic and obese mouse models faithfully recapitulate the pathogenesis of clinical metabolic OA. HFD feeding induces bone marrow inflammation and accelerated cartilage degeneration, while metformin reverses these pathological phenotypes via the regulation of lipid and prostaglandin metabolism in mice (48). Similarly, Type 2 diabetic rat models further confirm that metformin pretreatment counteracts hyperglycemia-triggered cartilage ultrastructural damage by suppressing oxidative stress and inflammatory mediators (53).

### 3.2 Rat cartilage injury models

Compared with mice, rats feature larger joint volumes, more convenient surgical manipulation and higher tolerance to local injection, rendering them ideal animal models for MIA chemical-induced OA modeling, cartilage defect repair and stem cell translational validation experiments. Current *in vivo* research in rats primarily focuses on autophagy-lysosomal regulation, biomaterial delivery, stem cell priming, RA and diabetic OA, with one notable contradictory outcome to be separately discussed below.

#### 3.2.1 Core chondroprotective and analgesic mechanisms

In MIA-induced rat OA models, metformin alleviates joint pain and cartilage degeneration by activating the autophagy-lysosomal pathway and regulating pain-associated mediators (37). Mechanistic investigation conducted by Xu T et al. has clarified that metformin restores the anabolic-catabolic balance of chondrocytes by inhibiting the overactivated PI3K/AKT/mTOR signaling pathway to normalize autophagic flux (54). In rat models of T2DM complicated with OA, metformin blocks hyperglycemia-induced oxidative stress and inflammatory signaling pathways to preserve the structural integrity of cartilage ultrastructure (53). In collagen-induced arthritis (CIA) rat models, systemic administration of metformin suppresses synovitis, osteoclastogenesis, cartilage matrix degradation and chondrocyte apoptosis, thereby inhibiting the systemic progression of inflammatory responses (55).

#### 3.2.2 Biomaterial sustained-release delivery systems for local therapy

Multiple types of composite drug carriers can enhance the therapeutic efficacy of metformin in rat OA models. Metformin hydrochloride-encapsulated strontium alginate hydrogel inhibits chondrocyte senescence and accelerates the repair of full-thickness cartilage defects (41). Dual-loaded nanohyalurosomes co-loaded with metformin and curcumin synergistically activate AMPK/TLR4-NF-κB signaling to improve joint mobility, relieve pain and restore cartilage architecture (56). PLGA porous microspheres co-loaded with metformin, KGN and PDGF-AB recruit endogenous mesenchymal stem cells, reprogram synovial macrophage polarization and reshape the anti-inflammatory intra-articular microenvironment to promote cartilage regeneration (57).

#### 3.2.3 Metformin priming enhances mesenchymal stem cell therapeutic capacity

Preconditioning with metformin enhances the anti-inflammatory and chondroprotective properties of adipose-derived mesenchymal stem cells (Ad-hMSCs). In rat OA models, metformin-stimulated Ad-hMSCs exert more robust pain relief and cartilage repair effects than untreated MSCs, providing a combinatorial cell therapy strategy for OA (58).

#### 3.2.4 Conflicting experimental outcome from DMM rodent models

Most studies conducted in mouse and rat models support the cartilage-protective efficacy of metformin. However, Ro DH et al. reported inconsistent results in DMM-operated rats, in particular intra-articular administration of metformin only induced thickening of the subchondral bone plate, and did not produce a significant inhibitory effect on cartilage degeneration (44). Potential sources of the aforementioned discrepancy are summarized as follows. First, heterogeneity exists in terms of species and model backgrounds. Mice and rats exhibit differences in skeletal development, baseline subchondral bone turnover rate and joint load distribution. Second, variations are observed across studies in drug administration regimens. Disparities exist in metformin injection concentration, administration frequency and local retention time in different investigations. Third, with respect to outcome evaluation, intervention duration and observation window differ across distinct studies.

### 3.3 Non-rodent animal models and toxicological evaluation of metformin

In addition to mice and rats, large animals (guinea pigs, pigs, rhesus macaques) and zebrafish, a commonly used toxicological model organism, have been employed to simulate age-related spontaneous OA, investigate primate physiological metabolism and conduct drug biosafety assessment, and prominent inconsistent conclusions across different research models have been clearly highlighted.

#### 3.3.1 Spontaneous age-related OA large animal models (guinea pig, pig)

Dunkin Hartley guinea pigs and aged pigs are utilized to model primary age-dependent OA in the absence of surgical intervention. The co-administration of rapamycin and metformin fails to alleviate spontaneous OA in guinea pigs, while rapamycin administered alone induces hyperglycemia and exacerbates joint degeneration (59). In aged pig models, rapamycin impairs skeletal muscle mitochondrial function, whereas metformin exhibits no detectable adverse effects on muscle mitochondrial homeostasis (60). These findings demonstrate that metformin cannot reverse rapamycin-mediated metabolic disturbance and age-related OA aggravation, and its regulatory effect on the muscle-joint axis is distinctly different from that of mTOR inhibitors.

#### 3.3.2 Non-human primate rhesus macaque OA models

As the preclinical model with the highest translational value that closely recapitulates human physiological metabolism, studies using male rhesus macaques with OA induced by partial medial meniscectomy (PMM) have verified that metformin activates AMPK signaling to decelerate the progression of cartilage degeneration, which is consistent with core conclusions drawn from small rodent studies (46). This evidence derived from non-human primate models strongly corroborates the clinical translational potential of metformin for OA treatment.

#### 3.3.3 Zebrafish toxicological evaluation of metformin

Zebrafish are established as standard aquatic toxicological model organisms that are widely utilized to assess the adverse reactions of metformin. Long-term exposure to ultra-high concentrations of metformin (50–10,000 µg/L) can induce cytoplasmic vacuolation in the erythrocytes of zebrafish (61). Nevertheless, the exposure doses adopted in these studies far exceed the clinical therapeutic blood concentration of metformin in humans, thus these observed toxic phenotypes cannot be extrapolated to the effect of routine clinical metformin administration. In clinical practice, oral metformin only induces mild transient gastrointestinal discomfort (nausea, diarrhea, vomiting) at standard doses, and does not cause severe toxicity to joints or systemic organs (62, 63). To minimize adverse reactions, individualized dose adjustment is still required according to patients’ age, liver and kidney function, and gastrointestinal tolerance.

### 3.4 Synthesis of consensus, conflicting results and unresolved research gaps from animal OA studies

#### 3.4.1 Unified consensus across all animal models

Activation of AMPK is the core conserved molecular mechanism underlying the chondroprotective activity of metformin in mice, rats and rhesus macaques, especially loss of AMPKα1 completely abrogates the therapeutic effects of metformin. The standard reproducible *in vivo* phenotypes include suppression of cartilage matrix degradation, inhibition of chondrocyte senescence, pyroptosis and ferroptosis, reversal of synovial infiltration of M1 macrophages, correction of aberrant subchondral bone remodeling, and alleviation of inflammatory joint pain. Local intra-articular delivery via cartilage-targeted biomaterials (nanocages, hydrogels, composite microspheres) significantly prolongs the joint retention time of metformin and improves its therapeutic efficacy, compared with oral administration or single free drug injection. When combined with bisphosphonates, exercise or stem cell-based therapy, metformin exerts synergistic therapeutic effects that are superior to single-drug intervention. Metformin at clinically equivalent doses exhibits acceptable systemic safety, and severe tissue toxicity only occurs at supraphysiological ultra-high concentrations in toxicological animal models.

#### 3.4.2 Primary conflicting outcomes and shared confounding drivers

Heterogeneous results from published animal studies can be categorized into three major groups. First, divergent cartilage protective effects in DMM rat models as aforementioned. Second, combined metformin and rapamycin treatment fails to produce therapeutic effects in spontaneous age-related guinea pig and pig OA models, while single-agent metformin alleviates surgically induced traumatic OA in rodents and primates. Third, varying degrees of regulatory effects on subchondral bone have been detected across different species and intervention cycles.

The common root causes of inter-study inconsistency may various. The most important thing is the different animal model types, such as surgically induced traumatic OA (DMM/ACLT/MIA), spontaneous age-related OA, metabolic OA (HFD/diabetes), RA combined with cartilage injury, non-human primate versus small rodent, etc. Also, the drug delivery strategies for metformin are different, particularly in oral gavage, single intra-articular injection of free metformin, sustained local release mediated by biomaterials. Another thing should be concerned is the intervention protocol, for instance, monotherapy versus combined treatment with bisphosphonates, exercise, stem cells or photothermal therapy. In addition, the bias in outcome assessment should be noticed, especially most of the research focus is restricted solely to subchondral bone, or studies lack comprehensive detection including cartilage histological scoring, inflammatory markers and matrix biomarkers. Last but not least, species-specific physiological differences remain concerns, especially the baseline metabolic level, bone turnover rate, joint immune microenvironment, and drug metabolic clearance capacity.

#### 3.4.3 Outstanding unresolved research gaps

In comparison to experiments conducted in mice and rats, studies of OA utilizing large animal models (including primates and pigs) remain extremely limited, and the inconsistent conclusions yielded by existing investigations on spontaneous age-related OA have yet to receive systematic mechanistic elucidation. To date, scarce head-to-head comparative studies have quantitatively evaluated the efficacy differences between systemic oral administration and local biomaterial-mediated delivery of metformin in the context of identical OA model backgrounds. Furthermore, the interactive regulatory network that connects systemic metabolism (i.e., obesity and diabetes), subchondral bone remodeling, synovial macrophage polarization, and chondrocyte ferroptosis/pyroptosis remains incompletely dissected *in in vivo systems*. Additionally, standardized and unified evaluation protocols for metformin-related *in vivo* OA research are currently absent, which leads to substantial heterogeneity in outcome indicators and poses considerable challenges for horizontal cross-study comparisons. Finally, the long-term safety and dose-effect relationship of repeated intra-articular injection of metformin-loaded carriers have not been systematically validated in preclinical animal models.

Consistent with observations from HFD-induced metabolic OA models, a growing body of preclinical evidence confirms that metformin exerts chondroprotective effects in the context of diet-induced metabolic dysfunction. Nevertheless, the therapeutic benefits of metformin are not universally observed, and appear to be tightly dependent on the host’s underlying metabolic milieu, particularly the presence and severity of insulin resistance and hyperglycemia. In contexts characterized by severe systemic metabolic disturbance, disrupted insulin signaling, chronic low-grade inflammation, and impaired AMPK activity in chondrocytes collectively create a permissive context that enables metformin to restore energy homeostasis and attenuate cartilage degeneration. In contrast, metformin typically exerts negligible chondroprotective effects when administered to OA models without overt insulin resistance or glycemic disorders, which partially accounts for the inconsistent outcomes reported across independent research laboratories. Although the protocol employing a single HFD regimen to induce metabolic OA reliably recapitulates diet-induced insulin resistance, it fails to enable differentiation of metformin’s efficacy across graded degrees of metabolic impairment. Future studies utilizing experimental models with stratified degrees of hyperglycemia and insulin resistance are therefore required to define the metabolic threshold necessary for metformin to exert joint protective effects, and to clarify whether this agent is unable to attenuate OA progression in normometabolic hosts.

Comprehensive profiling of cartilage matrix characteristics, subchondral bone microstructure, synovial macrophage polarization, and multiple programmed chondrocyte death markers is performed to systematically clarify the heterogeneous outcomes observed in previous animal studies. All representative animal studies investigating the cartilage-protective effects of metformin are summarized in **Table 2** to facilitate intuitive cross-model comparison.

**Table 2:** Summary of investigations of the effects of metformin on cartilage injury in animals.

Animals	Model types	Molding methods	Mechanisms	Refs
8-week-old male C57BL/6 mice	OA	Destabilization of the medial meniscus (DMM) operation	Attenuating cartilage degeneration in an experimental osteoarthritis model by regulating AMPK/mTOR	(29)
10-week-old male C57BL/6 mice	OA	DMM	Preventing cartilage degeneration and reducing pain behavior by modulating the AMPK signaling pathway	(26)
8-week-old male C57BL/6J mice	OA	Papain induction	Targeting and efficiently entering chondrocytes, further inducing prolonged intra-articular accumulation and achieving enhanced therapeutic efficacy for OA treatment	(34)
8-week-old C57BL/6 mice	OA	DMM	Attenuating cartilage degradation, activating autophagy and reducing apoptosis through activating AMPKα/SIRT1 signaling	(28)
3-week-old male C57BL/6 mice	OA	DMM	Showing stronger protective effects in HFD-fed mice than in normal diet (ND)-fed mice, reducing chondrocyte apoptosis and extracellular matrix degradation by inhibiting mTORC1 signaling and enhancing autophagy, reducing synovial macrophage infiltration and M1 polarization, reducing leptin levels in serum, articular chondrocytes, abdominal adipose tissue and infrapatellar fat pad in HFD-fed mice but not ND-fed mice	(24)
8-week-old male wild-type mice	OA	DMM	Inhibiting β-catenin^S552^ phosphorylation in articular cartilage in mice through activating AMPK signaling	(31)
8-week-old C57/BL6 male mice	OA	DMM	Reducing M1 polarization of synovial sublining macrophages and cartilage degeneration via PI3K/AKT pathways	(32)
10-week-old C57BL/6J male mice	OA	ACLT	Ameliorating OA progression, restoring joint bone remodeling, and diminishing inflammation in the joint micro environment by blocking the C-C motif chemokine ligand 2 (CCL2)/C–C motif chemokine receptor 2 (CCR2) signaling and reducing macrophage accumulation, synovial inflammation, and cartilage injury	(33)
4 to 6-week-old male DBA/1 mice	Rheumatoid arthritis (RA)	CIA	Exhibited potent anti-inflammatory effects by mediating macrophage polarization from M1 to M2	(23)
17-month-old male C57BL/6J mice	OA	Age-related formation	Attenuating the aging process of cartilage in aged mice, effectively alleviating cartilage degeneration in age-related OA, attenuating the pathogenesis of age-associated OA through iNOS/ONOO-/P53 signaling pathway	(35)
7-week-old male Wistar rats	OA	MIA-induced	Reducing pain, slowing OA progression by reducing bone and cartilage damage during OA development, reducing the levels of inflammatory mediators and catabolic factors in the synovium of OA rats, regulating inflammation-induced cell death in the joints of MIA-induced OA rats	(37)
10-week-old male C57BL6/J mice	OA	ACLT	Exerting anti-inflammatory properties by polarizing the macrophages from the M1 type (pro-inflammation) to the M2 type (antiinflammation), and pro-chondrocyte repair effect	(40)
Adult male Wistar rats (weighing 190–210 g)	Osteochondral defect	A circular hole (1 × 1 mm) was drilled in both the medial and lateral femoral condyle	The metformin/kartogenin-based cell-free osteochondral scaffold restored osteochondral defects completely.	(42)
12-week-old male Sprague–Dawley (SD) rats	Cartilage defect	A standard drill hole defect (1 mm wide and 1.5 mm deep) was made in the center site of the femoral trochlea	Accelerating cartilage repair and retarding the lesion-induced senescence of chondrocytes	(41)
4-month-old male Wistar rats	OA	DMM	Improving subchondral bone plate (SBP) thickening, but no effect on prevention of OA development	(44)
10-week-old wild-type and AMP-activated protein kinase (AMPK)α1 knockout (KO) mice, male rhesus macaques	OA	OA mice was induced with DMM surgery, and OA models of rhesus macaques was created with partial medial meniscectomy (PMM) operation.	Limiting OA development and delaying OA progression through AMPK phosphorylation	(46)
8–10-week-old outbred Institute of Cancer Research (ICR) (CD-2) female mice	OA	CIA	Attenuating the progression of OA by inhibiting the expression of receptor activator of nuclear factor kappa (RANK) and receptor activator of nuclear factor-κB ligand (RANKL) on osteoblasts and osteoclasts	(50)
3-month-old C57BL/6 mice	Estrogen deficiency and obesity models	Estrogen deficiency model was created with ovariectomy (OVX), obesity model was feed a high-fat diet (HFD)	Both metformin and exercise can alleviate OA, and the combination of the two methods showed a stronger effect in preventing cartilage degeneration.	(52)
8-week-old C57BL/6 male mice	OA	DMM	Decreasing cartilage degradation, improving subchondral bone remodeling, increasing cartilage matrix anabolism and inhibiting its catabolism, decreasing chondrocyte pyroptosis by inhibiting the activation of the NLRP3 inflammasome	(47)
8-week-old C57BL/6 male mice	OA	DMM	Attenuating cartilage degeneration ferroptosis induced by erastin, subchondral osteosclerosis, and subchondral bone angiogenesis in mice	(48)
3-month-old male C57/BL6 wild-type mice	OA	Induced by high-fat diet	Reducing prostaglandin (PG) level and cellular senescence in subchondral bone in mice treated with HFD, reducing the number of COX-2^+^ macrophages in subchondral bone, improving HFD-induced metabolic syndrome related OA, rescuing aberrant vessel and bone formation in HFD-treated mice, rescuing HFD-induced OA pain	(49)
8–10 weeks female ICR (CD-2) mice	OA	CIA	Decreasing levels of visfatin, cartilage biomarkers including C-terminal cross-linked telopeptide of type II collagen (CTX-II) and cartilage oligomeric matrix protein (COMP)	(51)
6-week-old male Wistar rats	OA	MIA	The metformin-stimulated adipose tissue–derived human mesenchymal stem cells (Ad-hMSCs) upregulated their immunoregulatory properties and downregulated their production of proinflammatory and catabolic mediators with autophagy induction, augmented chondroprotective and antinociceptive properties through attenuating the number of CGRP-immunoreactive neurons in lumbar 4 (L4) dorsal root ganglion (DRG)	(58)
7–8-week-old male Wistar rats	Collagen-induced arthritis (CIA)	CIA was induced in rats by intradermal injection of a mixture of bovine type II collagen and incomplete Freund’s adjuvant (IFA)	Inhibiting the progression of arthritis and systemic inflammation, inhibiting synovial hyperplasia, inflammatory infiltration, and neovascularization, alleviating the joint bone destruction, inhibiting cartilage destruction in CIA rats	(55)
Albino male rats weighing 170–200 g	T2DM	To induce diabetes, rats were fed on a high carbohydrate and fat diet (HCFD) for two weeks and then was given a single injection of streptozotocin (STZ)	Protecting against diabetes-induced loss of proteoglycan content of the articular cartilage, which partially protects against alterations to the chondrocyte and matrix ultrastructures induced by diabetes, inhibiting diabetes-induced hyperglycemia, glycated hemoglobin, inflammation, and oxidative stress	(53)
SD rats	OA	ACLT	Improving cartilage damage, inhibiting the catabolic metabolism in cartilage tissues, promoting the synthetic metabolism of cartilage tissue, inducing the activation of autophagy, inhibiting the activation of the PI3K/AKT/mTOR signaling pathway in the cartilage tissue of OA rats	(54)
Adult male SD rats weighing 200 ± 20 g	OA	MIA	Exerting anti-arthritic effect and halting OA progression through the potentiation of p-AMPK signaling and the suppression of its downstream TLR4/NF-κB signaling pathway	(56)
12-week-old male SD rats	OA	DMM	PMs@Met microspheres recruit intra-articular MSCs and encourage their development into chondrocytes, modulating the inflammatory joint environment by altering synovial macrophage polarization.	(57)
Dunkin Hartley guinea pigs	Not mentioned	Not mentioned	No effect on skeletal muscle mitochondria function in a model of age-related OA	(60)
Male Dunkin-Hartley guinea pigs	Age-related OA	Dietary rapamycin and metformin	Dietary rapamycin (14 ppm) and rapamycin+metformin (14 + 1000 ppm) treatment exacerbated the age-related progression of OA	(59)

## 4 Mechanistic integration and analysis of controversial points

**Figure 1:**
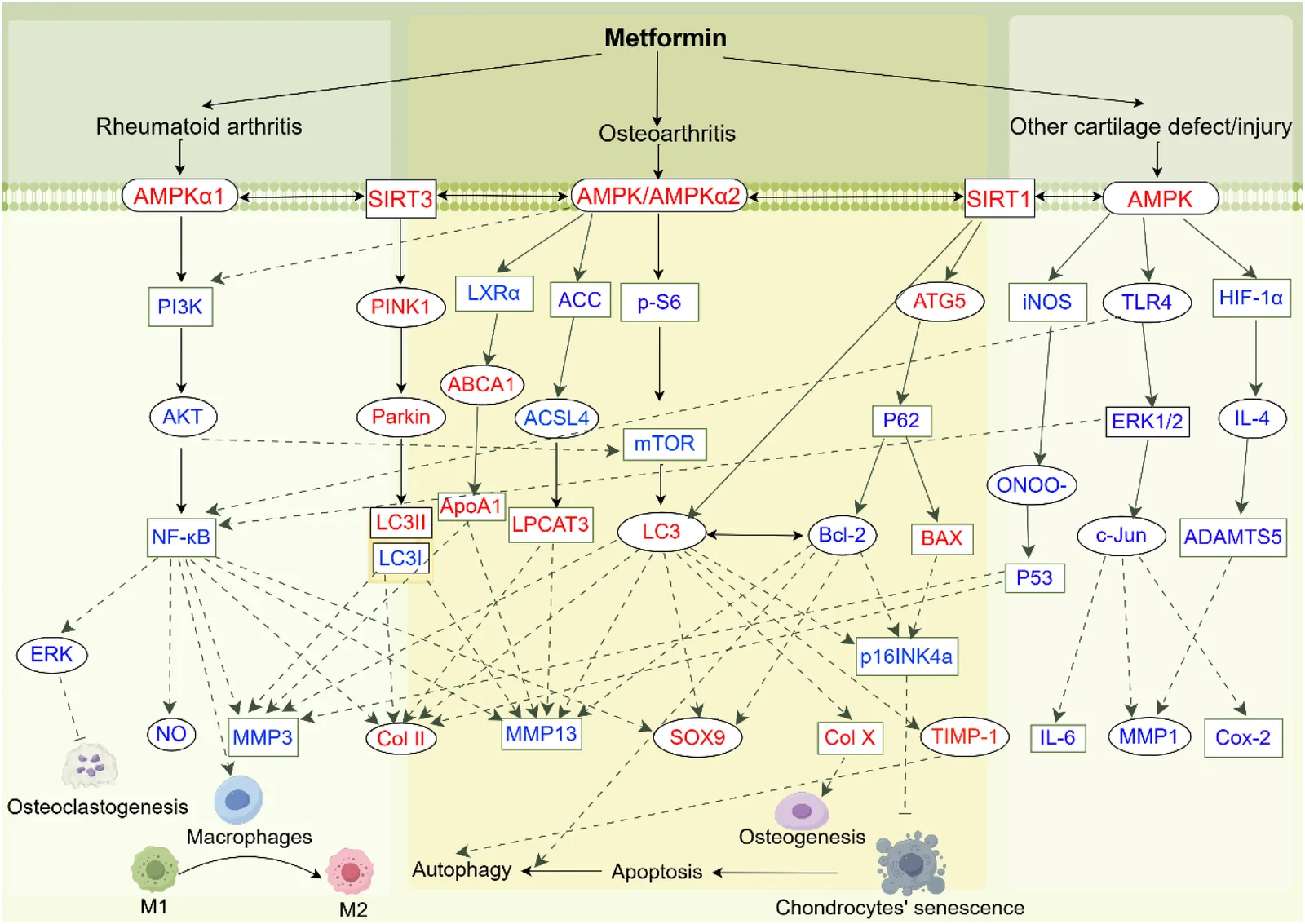
Summary diagram of key mechanism pathways. Metformin repairs cartilage defects/injuries due to OA, RA and other causes by upregulating those genes in red and downregulating the genes in blue. By Figdraw.

The regulatory effects of metformin on OA, RA and other cartilage defects or injuries universally converge on the activation of AMP-activated protein kinase (AMPK) as a shared molecular mechanism. However, owing to the distinct pathological characteristics of these diseases—OA characterized by cartilage degeneration, RA by autoimmune-driven inflammation, and cartilage injury by impaired posttraumatic repair—significant similarities and differences exist in their therapeutic effects, pathway emphasis, and experimental outcomes. Cellular models, including chondrocyte, fibroblast-like synoviocyte, and macrophage models, as well as animal models, such as rat OA, mouse CIA-induced RA, and pig osteochondral defect models, have consistently provided evidence for the AMPK-dependent anti-inflammatory and chondroprotective effects of metformin. In cellular systems, metformin exerts well-defined regulatory effects on single cell types, such as by inhibiting MMP-13 expression in chondrocytes. However, in animal models, the therapeutic efficacy of metformin is more substantially modulated by animal strain and modeling methodology because of complex interactions within systemic metabolic and immune networks. This is evidenced by the attenuated therapeutic outcomes observed in lean OA models compared with their obese counterparts. In cellular models of RA, metformin has been shown to have significant immunomodulatory effects on immune cells. Conversely, in RA animal models, higher dosages or extended treatment durations are required to achieve observable reductions in joint swelling. Furthermore, the amelioration of bone erosion in advanced RA models remains limited. These discrepancies may explain the divergent conclusions regarding the efficacy of metformin drawn from different experimental models. Studies by Ro DH et al. and others have concluded that “metformin lacks anti-aging or joint-protective effects,” a finding that contradicts the majority of published studies. This heterogeneous outcome cannot be attributed to a single factor and likely arises from multiple interrelated experimental variables. Apart from disparities in model design between their aging protocol and physiological aging trajectories, one plausible explanation involves inadequate drug target site exposure: oral metformin exhibits limited joint tissue bioavailability, and circulating drug concentrations may fail to reach therapeutically effective levels within avascular articular cartilage. Furthermore, the basal activation status of the AMPK pathway in chondrocytes varies across experimental systems, which determines cellular responsiveness to metformin. Importantly, this hypothesis remains speculative, as direct *in vivo* measurements linking intra-articular metformin concentration to divergent therapeutic outcomes are still lacking. Additional confounding variables include differences in administered dosage, the timing of treatment initiation relative to OA onset, total intervention duration, animal strain, OA induction strategy, and the terminal time point selected for cartilage phenotyping. When contrasting positive and negative preclinical reports, collective differences across these experimental parameters jointly shape inconsistent phenotypic observations, highlighting that metformin’s chondroprotective action is highly context-dependent (44). The therapeutic effects of metformin on joint diseases are mediated primarily through AMPK-dependent mechanisms, with select AMPK-independent pathways concurrently activated (64). AMPK-dependent mechanisms constitute the principal pathway mediating the therapeutic efficacy of metformin in OA, RA, and cartilage defects/injuries. Through the activation of AMPK phosphorylation, metformin modulates multiple downstream signaling pathways, including the inhibition of mTOR, NF-κB, and MAPK signaling (27, 30). Research concerning the impact of metformin on cartilage repair has shown that the effects of metformin are highly homogeneous. More in-depth, mechanism-specific individualized investigations are notably scarce. To provide a clearer representation of the key signaling pathways through which metformin regulates OA, RA, and cartilage injury, as discussed in this article, a comprehensive signaling pathway map is shown in **Figure 1**.

## 5 Evidence-based clinical effects of metformin on cartilage

Genetic differences between animal and human models determine differential pharmacological responses to therapeutic compounds. Consequently, novel pharmaceuticals must undergo rigorous clinical evaluation prior to market authorization to ensure both safety in treating specific diseases and achievement of the desired therapeutic efficacy. This clinical development paradigm is typically structured into three principal phases: phase I clinical trials assess drug safety and tolerability profiles; phase II clinical trials further evaluate therapeutic efficacy while continuing safety monitoring; and phase III clinical trials validate efficacy and safety within larger patient cohorts. Through this sequential investigational framework, researchers obtain comprehensive data essential for determining a drug’s suitability for broad clinical application.

### 5.1 Clinical research on the effect of metformin in cartilage injury

To further investigate the effects of metformin in OA patients, Na HS et al. evaluated structural progression within the knees of diabetic OA patients receiving metformin treatment using radiographic monitoring. Their results revealed a lower mean Kellgren and Lawrence (KL) grade in the metformin-treated group than in the non-metformin group, indicating that metformin inhibits the progression of OA, as indicated by radiographic monitoring, and demonstrates an anti-inflammatory effect in these patients (37). Lu CH et al. conducted a nationwide, retrospective, matched-cohort study in Taiwan utilizing data from patients diagnosed with OA and T2DM identified between 1 January 2000 and 31 December 2010 according to the International Classification of Diseases, Ninth Revision (ICD-9) - Clinical Modification (CM) codes 715.XX (OA) and 250.XX (T2DM). The results of this study revealed that patients with concomitant OA and T2DM who received combination therapy with COX-2 inhibitors and metformin had lower rates of joint replacement surgery than those who did not receive this combination did. This observed reduction may be attributable to the combination therapy demonstrating a significantly greater decrease in proinflammatory factors than therapy without metformin achieved (65). Wang Y et al. conducted an analysis of Osteoarthritis Initiative participants who had radiographic knee OA (Kellgren–Lawrence grade ≥ 2) and obesity [body mass index (BMI) ≥ 30 kg/m^2^]. Their findings indicated that metformin use was associated with a trend toward a reduced risk of total knee replacement over a 6-year follow-up period [adjusted odds ratio (aOR) = 0.30, 95% confidence interval (CI) 0.07–1.30, p = 0.11], following adjustment for age, sex, BMI, Kellgren–Lawrence grade, pain score, and self-reported diabetes. Collectively, these findings suggest that metformin use has a beneficial effect on long-term knee joint outcomes in individuals with concomitant knee OA and obesity (66). Abdallah MS et al. demonstrated that, compared with methotrexate monotherapy, the coadministration of metformin increased the antirheumatic efficacy of methotrexate in patients with RA, indicating that metformin serves as a beneficial adjuvant to methotrexate in patients with RA (67). Ruan G et al. described a study protocol for a randomized double-blind placebo-controlled trial to evaluate whether metformin can alleviate tibiofemoral cartilage volume loss and knee symptoms in overweight patients with knee osteoarthritis but did not provide conclusive findings (68). A retrospective study utilizing electronic health records retrieved from Hong Kong public primary care clinics led by Lai FTT et al. demonstrated that metformin use exhibited a protective effect against knee OA progression and reduced the risk of total knee replacement (TKR) among patients with diabetes (69). To investigate whether metformin use is associated with a reduced risk of total knee arthroplasty (TKA) and a reduced severity of knee pain in patients with knee OA and diabetes or obesity, Chen S et al. selected participants who were diagnosed with knee OA and diabetes or obesity between June 2000 and July 2019 from a local hospital’s information system. Analyses were performed utilizing log-binomial regression, linear regression, and propensity score weighting. The results indicated that metformin reduces the risk of TKA and alleviates knee pain severity in patients with metabolic-associated OA (70). To address the uncertainty regarding whether metformin use is associated with a reduced risk of joint replacement in patients with T2DM, Zhu Z et al. identified patients diagnosed with diabetes mellitus between January 2000 and December 2012 using the ICD-9 codes 250.xx from the database of Taiwan’s National Health Insurance Research Database. After evaluating the risks of TKR or total hip replacement (THR) using Cox proportional hazards regression, they concluded that metformin use significantly reduced the risk of total joint replacement in patients with T2DM (71). Alimoradi N et al. conducted a double-blind, placebo-controlled clinical trial to evaluate the efficacy of metformin versus placebo in overweight patients with knee OA. Their findings demonstrated that metformin significantly alleviated pain and improved the activities of daily living, sport and recreation, and quality of life in OA patients. Furthermore, OA susceptibility was closely associated with the wild-type genotype of Bcl-2 and the wild-type allele of CXCL-16 (72). In a large, nationwide cohort study of individuals with diabetes, Baker MC et al. reported a 24% reduction in the risk of developing OA among patients treated with metformin compared with those treated with sulfonylureas (73). Li F undertook a retrospective cohort analysis among elderly patients (aged ≥65 years) with hip OA and T2DM. This study demonstrated that metformin utilization was associated with a reduced risk of total hip arthroplasty (THA) in this patient population. Furthermore, this protective effect exhibited both cumulative and dose-dependent characteristics (74). Lim YZ et al. enrolled 102 community-dwelling participants who were diagnosed with symptomatic knee OA and concomitant overweight or obesity to investigate whether metformin reduces knee pain over a 6-month period (75). However, their results remain unpublished, precluding definitive conclusions. To investigate the efficacy of metformin as an adjuvant therapy for obese patients with knee OA, Aiad AAE et al. recruited 50 obese patients with knee OA and randomly assigned them to two groups: a placebo group (n=25) and a metformin group (n=25). Serum levels of cartilage oligomeric matrix protein (COMP), C-terminal cross-linked telopeptide of type I collagen (CTX-1), and IL-1β were measured. The Western Ontario and McMaster Universities Arthritis Index (WOMAC) was used to assess knee pain, stiffness, and physical function at baseline and after 12 weeks. These results support the conclusion that metformin may serve as a suitable adjuvant therapy for obese patients with knee OA, as it has beneficial effects on mitigating cartilage degradation and inflammation, as evidenced by significant reductions in serum COMP, CTX-1, and IL-1β levels (11). After 242 participants aged 54 to 77 years were enrolled, the participants were divided into four groups—control subjects (n=72), nondiabetic OA patients (n=58), metformin-treated OA diabetic patients (n=55), and OA diabetic patients receiving alternative antidiabetic agents (n=57)—Karim A et al. quantified serum zonulin levels (a biomarker of intestinal permeability), hand grip strength (HGS), the Oxford Knee score (OKS) to assess osteoarthritis severity, and short physical performance battery (SPPB) scores to evaluate physical function. Preclinical and observational evidence suggests that metformin may enhance HGS and physical performance by reducing zonulin concentrations, thereby preserving intestinal barrier integrity in OA patients (76). Building on a previous study, Alimoradi N et al. conducted a clinical trial to evaluate the potential molecular mechanisms underlying the efficacy of metformin in ameliorating OA symptoms (72). These results demonstrate that metformin may alleviate pain and inflammation in OA patients through modulation of the miR-451/CXCL16 signaling pathway and BCL-2. Furthermore, the analgesic effects of metformin are attributable to multiple mechanisms, including its anti-inflammatory and anti-aging properties (77). To evaluate the potential effects of metformin phonophoresis (MFPH) combined with exercise therapy (EX) versus MFPH alone or therapeutic ultrasound (US) alone on knee pain, function, and range of motion (ROM) in patients with chronic knee OA, Abed MS et al. conducted a randomized clinical trial. Comparisons of within-group and between-group effects for knee ROM and functional disability were performed using multivariate analysis of variance (MANOVA). Posttreatment outcomes demonstrated statistically and clinically significant improvements in pain intensity, knee ROM, and functional capacity in the MFPH group. Furthermore, compared with the other interventions, the combination of MFPH and exercise was superior in reducing knee OA symptoms (78). Although numerous studies have substantiated the role of metformin in cartilage repair, conflicting evidence has emerged. To examine whether metformin reduces the risk of OA, Barnett LA et al. conducted a cohort study utilizing data from the Consultations in Primary Care Archive, involving 3217 patients with T2DM. Following statistical analysis of the collected data, they reported no association between baseline metformin treatment and OA incidence, and no significant association was detected between cumulative exposure to metformin prescription over time and OA risk (79). To facilitate a better understanding of the current status of metformin research in cartilage injury clinical trials, summary of the relevant clinical studies is provided in **Table 3**.

**Table 3:** Summary of clinical studies on the effects of metformin on cartilage injury.

Type of study	Research model	Experiment time and site	Metformin usage	Findings	Refs
A retrospective study	41 OA patients (nondiabetic = 23 patients, diabetic with metformin=18 patients, all female).	Not clearly stated	Not clearly stated	Metformin inhibits OA clinical symptoms in OA patients with diabetes treated with celecoxib > 3 years.	(37)
A retrospective matched-cohort design	968 OA&T2DM patients with COX-2 inhibitor and metformin in case group; 1936 OA&T2DM patients with COX-2 inhibitor as control group.	2000.1.1-2010.12.31, Taiwan	Not clearly stated	Combination COX-2 inhibitors and metformin therapy in OA patients with T2DM are associated with lower joint replacement surgery rates than COX-2 inhibitor alone.	(65)
A prospective, randomized, double-blinded, placebo-controlled study	60 RA patients with metformin + MTX for 12 weeks; 60 RA patients with placebo + MTX for 12 weeks.	2019.08-2020.12, Menoufia University Hospital, Egypt	Placebo group received methotrexate (7.5 mg/week, intramuscular injection) plus one placebo tablet daily; metformin group received methotrexate (7.5 mg/week, intramuscular injection) plus XR metformin 1000 mg tablet once daily with food for 12 weeks.	Metformin improves the antirheumatic effect of methotrexate in RA.	(67)
A multicenter, randomized, double-blind, and placebo-controlled trial	262 overweight knee OA patients treated with metformin or placebo 1:1 for 24 months	2019-2021. Experiment sites: Guangzhou First People’s Hospital, Guangzhou, China; Sun Yat-sen Memorial Hospital of the Sun Yat-sen University, Guangzhou, China; Zhujiang Hospital of Southern Medical University, Guangzhou, China; Foshan First People’s Hospital, Foshan, China; Liwan Central Hospital of Guangzhou, Guangzhou, China; the Third People’s Hospital of Shenzhen, Shenzhen, China; the Second Affiliated Hospital of Nanchang University, Nanchang, China.	Start from 0.5 g/day for the first 2 weeks; increase to 1 g/day for the second 2 weeks; and further increase to 2 g/day for the remaining period if tolerated.	No clear conclusions are drawn.	(68)
A propensity-score-matched retrospective cohort study	93,330 diabetes patients (46,665 regular metformin users, 46,665 nonusers)	Visited during 2007 to 2010, were followed up from 2011 to 2014 to determine the incidence of total knee replacement (TKR). The experiment was conducted in Chinese University of Hong Kong.	Regular metformin users were defined as patients who had≥4 prescriptions over one year; patients who were not prescribed metformin in the past year were identified as nonusers.	Metformin shows a protective role against knee OA progression and decreases later TKR incidence among type 2 diabetic patients.	(69)
A retrospective cohort study	862 knee OA patients (346 metformin nonusers, 362 metformin users)	2000.6.10-2019.7.15, Zhujiang Hospital, Guangzhou, Guangdong, China	Regular metformin users: if they were recorded using metformin following the doctor’s advice or self-reported regular metformin use in past medical history and had been using metformin for at least 6 months. Nonusers of metformin: participants who were not recorded using metformin at physician orders or did not ever report the use of metformin or whose metformin was first administered after TKA or who used metformin for less than 6 months. Duration of metformin use using the median (2100 days) as the cutoff point and total metformin dose using the median (1048 g) as the cutoff point.	Metformin reduces degree of knee pain and risk of TKA in knee OA patients with diabetes.	(70)
A population-based matched cohort study	40694 T2DM patients (20347 were not treated with metformin and 20347 were treated with metformin)	2000.1-2012.12, Taiwan National Health Insurance Research Database	Participants were categorized into 3 groups based on daily dosage of metformin (0 g, < 1.0 g and ≥ 1.0 g) at 3, 12 and 24 months after the first prescription	Metformin reduces the risk of total joint replacement of T2DM patients.	(71)
A randomized double-blind placebo-controlled clinical trial	88 cases diagnosed with OA (44 cases receiving metformin, 44 cases receiving an identical inert placebo), 92 healthy non-OA participants were included as a negative control group	Shiraz University of Medical Sciences, Shiraz, Iran.	Receiving drug/placebo for 4 consecutive months (starting dose 0.5 g/day for the first week, increase to 1 g/day for the second week, and further increase to 1.5 g/day for the remaining period)	Metformin improves OA as indicated by KOOS score.	(72, 77)
A retrospective cohort study	41874 T2DM patients (20937 individuals used metformin, 20937 individuals used sulfonylurea)	2003.12.1-2019.12.31, from Optum deidentified Clinformatics Data Mart Database. Data were analyzed from April to December 2021 in Stanford University, Stanford, California, USA.	Metformin or sulfonylurea medication for at least 90 days.	Metformin reduces the risk of OA development but not joint replacement in T2DM patients.	(73)
A retrospective study	716 patients with hip OA and T2DM (308 metformin users, 308 metformin nonusers)	2012.1-2021.12, The First Affiliated Hospital of Harbin Medical University, Harbin, China.	The durations of metformin use were classified as short-term or long-term metformin use according to the median duration (1650 days). Participants were categorized into two groups on the basis of the daily dosage of metformin (low dose with <1.0 g and high dose with ≥1.0 g).	The ability of metformin to lower the risk of THA is cumulative and dose dependent in patients with hip OA and T2DM.	(74)
A randomized, double-blind, placebo-controlled trial	102 knee OA patients with obesity (51 receive metformin, 51 receive identical placebo)	2021.6.16-2023.9.30, Monash University, Melbourne, Victoria, Australia.	Treatment with study drug was initiated at a dose of 500 mg once a day with the evening meal. Over 6 weeks, the dose of study drug was titrated to 2000 mg once daily (or placebo once daily) to minimize gastrointestinal side effects.	No clear conclusions are drawn.	(75)
A randomized, double-blind, placebo-controlled study	50 obese knee OA patients (25 in metformin group, 25 in placebo group)	2021.2.1-2022.3.12, Tanta university hospital, Tanta, Egypt.	The metformin group (n=25) which was treated with metformin 500 mg orally bis in die (BID) plus celecoxib 200 mg orally once daily, and the placebo group (n=25) which was treated with placebo tablets BID plus celecoxib 200 mg orally once daily for 12 weeks.	Metformin may have beneficial effects on cartilage degradation and inflammation as an adjuvant therapy in patients with knee OA and obesity, as evidenced by the significant decreases in serum COMP, CTX-1 and IL-1β levels.	(11)
A prospective study	242 patients (control 72, OA nondiabetic 58, OA diabetic on metformin 55, OA diabetic on other anti-diabetics 57)	Rehman Medical Institute, Peshawar, Pakistan	Not clearly stated.	Metformin improves hand-grip strength and physical performance, as well as reduces zonulin levels.	(76)
A parallel-group, randomized, single-blinded clinical trial	78 knee OA patients (metformin phonophoresis gel + exercise therapy 26, metformin phonophoresis gel 26, nondrug acoustic gel 26)	Cairo University, Cairo, Egypt	Metformin HCl powder 34.5 g was dissolved in 20 ml glycerin. Then, the prepared solution was added gradually to 2850 g of the ultrasound gel with constant mixing to ensure homogeneity. Finally, 2.85 g of sodium citrate was added as a preservative, and the mixing continued until clear gel was obtained (10–15 min).	Metformin phonophoresis combined with exercise treatment may benefit patients with KOA by improving knee pain, mobility, and functional disability management.	(78)
An electronic health record cohort study	3217 T2DM patients (metformin Group 1838, nonmetformin Group 1379) had no prior OA record.	2002.1-2003.12, Consultations in Primary Care Archive (CiPCA) database, an anonymized database of routinely recorded information from 13 general practices in North Staffordshire, UK.	Not clearly stated.	This study has not identified evidence of an association of metformin treatment at baseline and OA.	(79)

### 5.2 Limitations of clinical evidence and future research directions

Currently, clinical investigations into the therapeutic application of metformin for cartilage repair remain scarce, predominantly retrospective analyses and few prospective trials. More importantly, most of these studies indicated that the protective effects of metformin on OA occur in patients with T2DM or obesity. Consequently, the available clinical evidence is limited. Furthermore, the majority of existing studies are small-scale, short-term observational investigations that often exhibit methodological limitations and susceptibility to bias. These methodological limitations include insufficient sample sizes and follow-up durations, inadequate standardization of trial protocols, inconsistent intervention strategies, and incomplete outcome assessment frameworks. Potential sources of bias include selection bias, information bias, confounding bias, and publication bias. Future research should incorporate multicenter, large-scale, placebo-controlled prospective studies to robustly establish the clinical efficacy of metformin across diverse joint pathologies. Stratified analyses or subgroup investigations should be conducted to delineate variations in therapeutic response among patients with differing metabolic statuses, injury severities, and age demographics. Dedicated dose-finding trials are warranted to determine the optimal dosage and duration of metformin administration for both therapeutic efficacy and safety. A standardized evaluation system should be established that incorporates a unified assessment framework for cartilage repair and joint function. This framework must integrate subjective symptom scores, objective imaging indicators (e.g., magnetic resonance observation of cartilage repair tissue [MOCART] score), histological assessments (such as cartilage repair quality scores), and molecular biomarkers (including matrix metalloproteinases [MMPs] and type II collagen degradation products) to enable a comprehensive and objective evaluation of therapeutic outcomes.

The promising chondroprotective effects of metformin identified in *in vitro* experiments and HFD-fed animal models provide critical mechanistic insights into metabolic OA. However, these preclinical findings are not directly generalizable to human populations. It must be emphasized that the existing evidence base from human studies remains markedly limited. The majority of available clinical evidence is derived from retrospective observational database analyses, while adequately powered prospective investigations and randomized controlled trials are still lacking. In addition to the inherent confounding risks within real-world datasets, interspecies variations in cartilage biology, drug metabolism, and disease progression further impede the straightforward translation of findings from animal models to clinical practice. Therefore, any therapeutic inference derived from preclinical work requires cautious interpretation, and definitive clinical recommendations await further high-quality human investigations.

Notably, most available clinical evidence originates from retrospective observational analyses based on administrative databases such as NHIRD and Optum, which are inherently susceptible to confounding by indication. Compared with non-metformin users, individuals receiving metformin generally differ systematically in metabolic profiles, lifestyle patterns, comorbid conditions and medication histories. Although propensity score matching and multivariate adjustment are widely adopted to balance measurable covariates, residual unmeasured confounding cannot be completely ruled out. Accordingly, the observed associations between metformin use and favorable OA outcomes must be interpreted with caution, and cannot be directly recognized as causal evidence. Well-designed prospective cohorts and randomized controlled trials remain necessary to validate the clinical efficacy of metformin against metabolic OA.

## 6 Other investigations

As an emerging interdisciplinary field integrating biology and informatics, bioinformatics elucidates the mechanisms and principles governing life, advances biological research, and furnishes novel insights and methodologies for life science applications. To evaluate the impact of metformin targets on OA risk, Zhang Y et al. conducted a Mendelian randomization (MR) study. They reported that genetically predicted AMPK expression was inversely associated with OA at any anatomical site and that higher levels of genetically predicted growth differentiation factor 15 (GDF-15) were associated with a reduced risk of hip OA. Collectively, these findings indicate that AMPK and GDF-15 are promising therapeutic targets in OA, particularly hip OA (80). By employing a comprehensive analytical pipeline integrating two-sample MR (utilizing genetic proxies for antidiabetic drug targets), summary-based MR (for mRNA expression), and colocalization analysis (for drug-target genes) to evaluate causal relationships with twelve OA phenotypes, Fu et al. reported that sulfonylurea targets ABCC8 and KCNJ11 were associated with an increased risk of OA at any site, whereas PPARG was associated with a decreased risk of hand, finger, and thumb OA. Metformin and glucagon-like peptide-1 receptor agonists (GLP1-RAs), which target GPD1 and GLP1R, respectively, were associated with a reduced risk of knee and finger OA. In summary-based MR analyses, the expression levels of KCNJ11, GANAB, ABCA1, and GSTP1—genes targeted by antidiabetic drugs—demonstrated significant associations with at least one OA phenotype, with these findings replicated across a minimum of two independent gene expression datasets. Furthermore, increased expression of KCNJ11 was associated with decreased OA risk, and KCNJ11 colocalized with at least one OA phenotype. Collectively, the results of this study suggest that certain antidiabetic drugs may have therapeutic potential for treating cartilage injury because they share molecular targets that are involved in OA pathogenesis (81). Metformin, an established agonist of AMPK, has diverse pharmacological effects. However, the specific receptor subtype that it activates in OA and RA remains undefined. Research by Chen Y et al. revealed significantly higher AMPKα1 expression in the synovium of RA patients than in that of OA patients on the basis of NCBI GEO profiles and RNA sequencing results. These findings indicate that metformin primarily activates AMPKα1, suggesting that metformin may exhibit greater therapeutic efficacy in RA than in OA (82).

## 7 Conclusions and outlook

Within the current management of OA, RA and other cartilage defects/injuries, both nonsteroidal anti-inflammatory drugs (NSAIDs) and traditional Chinese medicine (TCM) have demonstrated therapeutic efficacy. Although NSAIDs can be administered through multiple routes, their long-term utilization necessitates the rigorous monitoring of gastrointestinal and cardiovascular function. Certain formulations require concomitant food intake to mitigate gastrointestinal irritation, thereby complicating medication adherence for patients. TCM preparations are predominantly administered as decoctions, pills, or topical patches. Decoction preparation is time-consuming, but topical patches may elicit allergic reactions in the skin. Furthermore, specific TCM agents incur significant costs, and protracted use may impose a substantial economic burden on patients. In contrast to NSAIDs and TCM, metformin has a more precise mechanism of action, directly targeting the pathological core of osteoarthritis and concurrently exerting dual anti-inflammatory and chondroprotective effects. It has a superior safety profile, characterized by favorable tolerability during extended administration and a lower incidence of adverse reactions. Metformin also offers the advantage of multimodal therapeutic effects, enabling the management of comorbidities and addressing complex clinical requirements. Metformin is administered orally, primarily in tablet form, which ensures convenient administration and obviates the need for special preparation, thereby promoting high patient compliance. As a generic medication, metformin benefits from a well-established production process and low acquisition cost. Consequently, its long-term economic burden is significantly lower than that of certain TCM agents and newer NSAIDs, rendering it particularly suitable for chronic management in primary care settings and diverse patient populations. Additionally, metformin dosage can be individually titrated on the basis of patient-specific factors such as age, body weight, and disease severity, increasing its clinical flexibility and adaptability.

Conventional metformin formulations are characterized by low bioavailability and suboptimal tissue targeting. Upon oral administration, limited drug accumulation at osteochondrosis lesion sites compromises the full realization of its therapeutic potential. Nanotechnology presents a promising strategy to address this limitation (83). Accumulating evidence has demonstrated that nanoformulations significantly increase the bioavailability, tissue targeting, and therapeutic efficacy of metformin in cartilage-related pathologies, thereby increasing its prospects for clinical translation. In an MIA-induced osteoarthritis rat model, El-Haddad ME et al. reported that metformin-curcumin-coloaded nanohyaluronic acid nanoparticles inhibited OA progression and ameliorate articular cartilage structural degeneration, potentially through enhancing p-AMPK signaling (56). Through comprehensive *in vitro* cellular assays and *in vivo* animal investigations, Da W et al. found evidence substantiating that metformin-loaded tannic acid nanoparticles mitigate osteoarthritic cartilage degeneration. This therapeutic effect is mediated by the induction of mitochondrial biogenesis within chondrocytes, which promotes mitochondrial homeostasis and thus attenuating the progression of OA (83). El-Haddad ME et al. demonstrated that the intra-articular administration of metformin-curcumin cationic PLGA nanoparticles restored joint structure in a MIA-induced OA model through coordinated modulation of the crosstalk among the miR-93, TNFAIP3/TLR/NF-κB, and AMPK/SIRT1 signaling pathways (84). Therefore, metformin nanoformulations represent promising experimental or preclinical strategies but require further translational and clinical validation. Since 3D-printed scaffolds are cutting-edge strategies for bone and osteochondral repair (85, 86), in particular, metformin-loaded 3D-printed hydrogels have demonstrated potential in bone regeneration (87), and incorporating metformin into such scaffolds may emerge as a promising future approach for treating osteochondral and cartilage injuries. Two primary delivery strategies for metformin, namely oral administration and nanocarrier-assisted intra-articular injection, have been investigated for OA treatment, and substantial discrepancies exist in their translational prospects. Oral administration of metformin exhibits multiple distinct advantages including mature clinical application, confirmed long-term safety, convenient administration and high patient compliance, rendering it particularly applicable to metabolic OA patients with comorbid insulin resistance, obesity and other systemic metabolic disorders. Nevertheless, this route is constrained by the limited bioavailability of metformin within avascular articular cartilage, which may restrict the achievement of effective local therapeutic concentrations. In comparison, intra-articular injection achieves targeted drug enrichment in joint tissue and reduces systemic drug exposure. However, its translational progression is hindered by invasive puncture, poor patient acceptance of repeated injections, as well as persistent unresolved concerns regarding the biocompatibility, degradability and formulation standardization of nanocarriers. Collectively, these two approaches are not mutually competitive alternatives. Oral metformin targets both systemic metabolic dysfunction and joint lesions, while local intra-articular therapy focuses on alleviating isolated cartilage degeneration. Rational selection of administration route should be based on the metabolic phenotype and clinical OA manifestations of individual patients. These strategies represent a promising avenue for developing safer and more effective treatments for patients with cartilage damage, particularly in the case of comorbid metabolic disorders, thus advancing comprehensive improvements in the diagnostic and therapeutic management of cartilage-related diseases.

This review comprehensively summarizes *in vitro* experiments, animal studies, clinical research, and other investigations concerning the application of metformin in the treatment of cartilage injury. *In vitro* experiments primarily explore mechanisms and functions at the cellular level. Animal studies evaluate drug effects, toxicology, physiology, and other systemic biological responses. Clinical research encompasses medical trials conducted on human subjects to assess the safety, efficacy, and effectiveness of novel drugs, medical devices, or therapeutic interventions. Other investigations, such as bioinformatics analyses, primarily seek to elucidate drug mechanisms and targets through computational methodologies. While the majority of studies report positive outcomes for metformin in cartilage injury, a minority present negative conclusions. Metformin is a recognized AMPK activator, and most studies attribute its downstream effects to this receptor, although this perspective is clearly not comprehensive. Current clinical research predominantly consists of retrospective studies, and prospective studies involve limited case numbers. Consequently, larger-scale, randomized, prospective, controlled clinical trials are warranted to definitively establish the role of metformin in cartilage injury. In summary, metformin is a natural product–derived drug with emerging potential across multiple fields, necessitating further mechanistic exploration and clinical validation.

Future translational investigations are warranted to address the key outstanding questions regarding metformin treatment for metabolic OA. First, adequately powered, appropriately designed randomized controlled trials are indispensable to validate clinical efficacy and overcome confounding biases inherent in retrospective database studies. Second, further research should focus on identifying the optimal dosage and treatment duration of metformin, and comparing the merits and limitations of oral administration versus local intra-articular delivery. Third, stratified patient selection warrants greater academic attention: research participants should be grouped by metabolic profiles (insulin resistance, obesity, hyperglycemia) and OA severity to distinguish responders from non-responders and achieve precision intervention. In parallel, preclinical studies utilizing models with graded metabolic dysfunction will help clarify the specific metabolic conditions under which metformin exerts chondroprotective effects, laying a solid methodological foundation for subsequent clinical translation.
